# Supplementation of nitric oxide and spermidine alleviates the nickel stress-induced damage to growth, chlorophyll metabolism, and photosynthesis by upregulating ascorbate–glutathione and glyoxalase cycle functioning in tomato

**DOI:** 10.3389/fpls.2022.1039480

**Published:** 2022-10-27

**Authors:** Cheng Qin, Jie Shen, Mohammad Abass Ahanger

**Affiliations:** ^1^ Department of Life Sciences, University of Changzhi, Changzhi, China; ^2^ College of Life Science, Northwest A&F University, Xianyang, Shaanxi, China

**Keywords:** antioxidants, glyoxalase, oxidative stress, nickel, nitric oxide, spermidine

## Abstract

Experiments were conducted to evaluate the role of exogenously applied nitric oxide (NO; 50 µM) and spermidine (Spd; 100 µM) in alleviating the damaging effects of Ni (1 mM NiSO_4_6H_2_O) toxicity on the growth, chlorophyll metabolism, photosynthesis, and mineral content in tomato. Ni treatment significantly reduced the plant height, dry mass, and the contents of glutamate 1-semialdehyde, δ-amino levulinic acid, prototoporphyrin IX, Mg–prototoporphyrin IX, total chlorophyll, and carotenoids; however, the application of NO and Spd alleviated the decline considerably. Supplementation of NO and Spd mitigated the Ni-induced decline in photosynthesis, gas exchange, and chlorophyll fluorescence parameters. Ni caused oxidative damage, while the application of NO, Spd, and NO+Spd significantly reduced the oxidative stress parameters under normal and Ni toxicity. The application of NO and Spd enhanced the function of the antioxidant system and upregulated the activity of glyoxalase enzymes, reflecting significant reduction of the oxidative effects and methylglyoxal accumulation. Tolerance against Ni was further strengthened by the accumulation of proline and glycine betaine due to NO and Spd application. The decrease in the uptake of essential mineral elements such as N, P, K, and Mg was alleviated by NO and Spd. Hence, individual and combined supplementation of NO and Spd effectively alleviates the damaging effects of Ni on tomato.

## Introduction

In recent years, rapid industrial progress has raised many serious issues including heavy metal pollution, thereby imposing serious threats to livelihood. Among the key contributors of heavy metal pollution are the processes of smelting, mining, electroplating, burning of fossils, and phosphate fertilization (Salt and Kramer, 2000). Heavy metals are continuously disposed and added to the environment, rendering the surroundings unsafe for humans and crops. Excess accumulation of heavy metals seriously affects soil health and renders it less fertile, thereby affecting crop growth and productivity (Ahmad and Ashraf, 2011; Hassan et al., 2019). Nickel (Ni) is one of the metal pollutants with serious influence on the ecosystem and on human and plant health (Duda-Chodak and Baszczyk, 2008). Ni is a natural component of water and soil, but occurs at very low concentrations (McIlveen and Negusantil, 1994; McGrath, 1995). Excess concentrations of Ni can prove toxic and result in damaging alterations in the metabolism and growth of plants. Reduced plant growth due to Ni toxicity has been attributed to the significant reduction in photosynthesis, enzyme activity, membrane stability, and mineral nutrition (Kazemi et al., 2010; Khan and Khan, 2014). Metal stress-induced growth retardation is mainly attributed to the oxidative damage triggered by the excess accumulation of toxic reactive oxygen species (ROS) and methylglyoxal (MG), which severely affects the redox homeostasis, membrane function, and protein stability, among others (Shahid et al., 2014; Berni et al., 2019; Ahmad et al., 2020). In addition, the stress-induced decline in chlorophyll metabolism significantly contributes to overall growth alterations (Dalal and Tripathy, 2012; Qin et al., 2020). Plants upregulate their tolerance mechanisms to alleviate the damaging effects of ROS, and included in these key tolerance mechanisms are the antioxidant system, glyoxalase system, and osmolyte accumulation (Ahmad et al., 2020). The enzymatic and non-enzymatic components of the antioxidant system work in close coordination to maintain the cellular ROS concentrations, and they also contribute to redox homeostasis, maintenance of enzyme activity, and photosynthesis (Begum et al., 2020; Qin et al., 2021). The glyoxalase system consists of two key enzymes, i.e., glyoxalase I (Gly I) and glyoxalase II (Gly II), that act on MG to prevent its cytotoxic effects (Ahmad et al., 2020).

Nitric oxide (NO) is an endogenous signaling molecule that has been proven to be a ubiquitous molecule involved in regulating an array of physiological, biochemical, and molecular processes from germination to stress signalling and tolerance (Ahmad et al., 2018; Ahmad et al., 2020). The impact of NO produced endogenously or applied exogenously depends on its concentration and the site of production determining its beneficial or deleterious effect (Fatma et al., 2016; Asgher et al., 2017). At optimal concentrations, NO forms a key component of the signaling network, leading to the modulation of key physiological and biochemical pathways for better stress tolerance (Fatma et al., 2016; Soliman et al., 2019; Shang et al., 2022). Due to its unique chemical properties and biological action, NO has been considered as either a stress-inducing (Gould et al., 2003) or a protective agent (Hsu and Kao, 2004; Bai et al., 2015). NO-derived molecules, also known as reactive nitrogen species (RNS), play key roles in maintaining the intracellular redox homeostasis and signaling for the activation of antioxidant mechanisms. It has been reported that the application of NO in plants under stressful conditions helps protect the growth, photosynthesis, and enzyme activity by upregulating the tolerance mechanisms (Fatma et al., 2016; Nabi et al., 2019; Singhal et al., 2021). Recently, in *Hordeum vulgare* L., the application of NO has been reported to alleviate the damaging effects of copper on plant growth and photosynthesis by upregulating the antioxidant function and maintaining the redox homeostasis (Massoud et al., 2022).

Spermidine (Spd) is one of the polyamines and has been reported to actively participate in the growth and cellular function regulation in plants under normal and adverse conditions (Chen et al., 2019; Rakesh et al., 2021). Polyamines are low-molecular-weight nitrogen-containing molecules that are produced during metabolism in almost all types of cells (Chen et al., 2019). They have strong capacity to bind DNA, RNA, and proteins, and the accumulation of polyamines, including spermine, Spd, and putrescine, has been reported to occur under stress conditions, which is associated with the upregulation of their biosynthetic pathway (Hu et al., 2016; Bano et al., 2020). Polyamines play key roles in the alleviation of the oxidative damage to plants through their active role in upregulating the tolerance mechanisms aimed to neutralize toxic ROS (Minocha et al., 2014). Treatment with polyamines has been reported to alleviate the deleterious effects of water (Hassan et al., 2018) and heat stress-induced (Jing et al., 2020) oxidative damage by improving the activity of antioxidant enzymes and the osmolyte accumulation. However, reports on the interactive effects of NO and polyamines are rare.

Tomato (*Solanum lycopersicum* L.) is an important crop grown worldwide. It has rich antioxidative and anticancer activities ascribed to the presence of key metabolites including lycopene and carotene, among others. A considerable increase in soil Ni concentrations can adversely influence the growth and productivity of tomato, and the accumulation of Ni in edible fruit can affect humans. In this backdrop, it was hypothesized that foliar treatment with NO and Spd (individual and combined) can alleviate the Ni stress-induced alterations in the growth, chlorophyll metabolism, and mineral uptake by upregulating the ascorbate–glutathione cycle, glyoxalase cycle, and osmolyte accumulation. The growth, chlorophyll metabolism, photosynthesis, oxidative stress parameters, and tolerance mechanisms were calculated.

## Material and methods

Seeds of tomato (*S. lycopersicum* L. cultivar Dongfeng-199) were sterilized with 0.001% HgCl_2_ for 5 min, followed by thorough washing with distilled water. The sterilized seeds were sown in trays filled with a nutrient-rich peat-based substrate (Pindstrup, Ryomgård, Denmark). Five days after germination, the seedlings were transplanted into pots (diameter, 20 cm) filled with acid-washed sand and were regularly irrigated with full-strength Hoagland nutrient solution (200 ml per pot) on alternate days. Fifteen days after successful seedling establishment, the pots were divided into two groups: one group irrigated with normal Hoagland solution and another group irrigated with modified Hoagland solution containing 1 mM Ni (NiSO_4_6H_2_O). However, treatments with NO [in the form of 50 µM sodium nitroprusside (SNP)] and Spd (100 µM Spd trihydrochloride; Sigma-Aldrich, St. Louis, MO, USA) were given foliarly (10 ml per pot) with a hand sprayer using teepol as a surfactant. Detailed experimental treatments are as follows: a) control; b) Ni; c) 50 µM NO; d) 100 µM Spd; e) NO+Spd; f) Ni+NO; g) Ni+Spd; and h) Ni+NO+Spd. Treatments with Ni, NO, and Spd were given for another 15 days; therefore, analysis of the different parameters was done on 30-day-old plants. Pots were arranged in a completely randomized block design with four replicates for each treatment in a greenhouse with day/night temperatures of 30°C/25°C and relative humidity of 70 ± 5%. The different physiological and biochemical parameters determined included photosynthesis, oxidative stress markers, and the antioxidant and glyoxalase systems.

### Estimation of pigments, photosynthetic gas exchange parameters, and PSII function

The contents of total chlorophyll and carotenoids were estimated in accordance with the method of Arnon (1949). After homogenizing 100 mg fresh leaf in 80% acetone using a pestle and mortar, the extract was centrifuged and the optical density of the supernatant determined at 480, 645, and 663 nm. Gas exchange parameters such as the net photosynthesis (*P*_n_), stomatal conductance (*g*_s_), intercellular CO_2_ concentration (*C*_i_), and transpiration rate (*E*) were recorded using the LI-6400 photosynthesis system (LI-COR, Lincoln, NE, USA) between 0900 and 1200 hours. The modulated chlorophyll fluorometer PAM-2500 (Heinz Walz, Effeltrich, Germany) was used to calculate the chlorophyll fluorescence parameters including photosystem II (PSII) activity (*F*_v_/*F*_m_), photochemical quenching (qP), non-photochemical quenching (NPQ), and electron transport rate (ETR) after dark adapting the leaves for 25 min.

### Estimation of glutamate-1-semialdehyde, δ-amino levulinic acid, prototoporphyrin IX, Mg-prototoporphyrin IX, and protochlorophyllide

The glutamate-1-semialdehyde (GSA) content was measured as described by Kannangara and Schouboe (1985), and absorbance was taken at 620 nm after incubating the tissue with gabaculine. The content of δ-amino levulinic acid (δ-ALA) was estimated according to Harel and Klein (1972), and optical density was read at 555 nm after incubating the samples for 4 h with levulinic acid. For estimation of the contents of prototoporphyrin IX (Proto IX), Mg-prototoporphyrin IX (Mg-Proto IX), and protochlorophyllide (Pchlide), the method of Hodgins and Huystee (1986) was followed. Briefly, 300 mg of fresh leaf samples was extracted in 5 ml alkaline acetone and the optical density read at 575, 590, and 628 nm.

### Estimation of proline and glycine betaine

The proline content was determined by homogenizing the dry powdered sample in 3% sulfosalicylic acid using a pestle and mortar. The homogenate was centrifuged at 3,000 × *g* for 20 min and 2 ml supernatant mixed with glacial acetic acid and ninhydrin reagent. The resultant mixture was incubated at 100°C for 1 h. Thereafter, the samples were cooled in an ice bath, proline was separated using toluene, and the optical density was read at 520 nm (Bates et al., 1973). For the estimation of glycine betaine (GB), the method of Grieve and Grattan (1983) was followed. Dry samples were extracted in distilled water, and the extract was filtered and diluted by the addition of 2 N H_2_SO_4_. An appropriate aliquot of the diluted extract was mixed with cold KI–I_2_ reagent, followed by centrifugation at 10,000 × *g* for 15 min. The periodide crystals formed were dissolved in 1,2-dichloroethane and the optical density taken at 365 nm. The standard curve of GB was used for calculation.

### Measurement of lipid peroxidation, hydrogen peroxide, and activity of lipoxygenase

Lipid peroxidation was measured following the method of Heath and Packer (1968). Of the fresh leaf tissue, 100 mg was macerated in 1% trichloroacetic acid (TCA) and the extract centrifuged at 10,000 × *g*. The supernatant (1.0 ml) was reacted with 4 ml thiobarbituric acid for half an hour at 95°C. After cooling the samples in an ice bath, centrifugation was done at 5,000 × *g* for 5 min and the absorbance measured at 532 and 600 nm (Heath and Packer, 1968). Lipid peroxidation was expressed as the amount of malondialdehyde (MDA) formed. The hydrogen peroxide content was estimated by homogenizing fresh tissue in 0.1% TCA. After centrifugation at 12,000 × *g*, 0.5 ml of the supernatant was mixed with potassium phosphate buffer (pH 7.0) and potassium iodide. Absorbance was then taken at 390 nm, and the standard curve of H_2_O_2_ was used for calculation (Velikova et al., 2000). The method described by Doderer et al. (1992) was used to assay the activity of lipoxygenase (LOX; EC 1.13.11.12), and absorbance was taken at 234 nm. Linoleic acid was used as the substrate, and the extinction coefficient of 25 mM^−1^ cm^−1^ was used for calculation.

### Activity of glyoxalase I and glyoxalase II and content of methylglyoxal

Fresh tissue was extracted in cold 50 mM potassium phosphate buffer (pH 7.0) containing 10 mM KCl, 1 mM ascorbate, 1 mM β-mercaptoethanol, and 10% glycerol. The homogenate was centrifuged at 11,500 x g for 15 minutes at 4°C and the supernatant used as the enzyme source for assaying the activities of Gly I (EC 4.4.1.5) and Gly II (EC 3.1.2.6). The method described by Hasanuzzaman et al. (2011) was employed to estimate the activity of Gly I. A change in the optical density was noticed at 240 nm. For calculation, an extinction coefficient of 3.37 mM^−1^ cm^−1^ was used. For assay of the Gly II activity, the optical density was recorded at 412 nm and an extinction coefficient of 13.6 mM^−1^ cm^−1^ was used for calculation, in accordance with Principato et al. (1987). The content of MG was estimated according to Wild et al. (2012). After extracting leaf tissue in perchloric acid and centrifuging the homogenate for 10 min at 11,000 × *g*, the supernatant was reacted with sodium dihydrogen phosphate and *N*-acetyl-l-cysteine. After 10 min, the *N*-α-acetyl-*S*-(1-hydroxy-2-oxo-prop-1-yl)cysteine formed was read at 288 nm.

### Assay of the antioxidant enzymes and contents of ascorbate and reduced glutathione

Antioxidant enzymes were extracted by homogenizing 1.0 g fresh leaf tissue in 100 mM cold phosphate buffer (pH 7.8) containing 1% polyvinylpyrrolidone (PVP), 1 mM EDTA, and 0.1 mM phenylmethylsulfonyl (PMSF) using a pre-chilled pestle and mortar. After centrifuging the homogenate at 12,000 × *g* for 15 min at 4°C, the supernatant was used as the enzyme source. The activity of superoxide dismutase (SOD; EC 1.15.1.1) was assayed following Bayer and Fridovich (1987), and absorbance was taken at 560 nm after incubating the samples for 15 min. The activity of ascorbate peroxidase (APX, EC 1.11.1.11) was measured according to Nakano and Asada (1981) and the absorbance taken at 290 nm for 3 min; for glutathione reductase (GR; EC 1.6.4.2), the method of Foyer and Halliwell (1976) was used and absorbance was taken at 340 nm for 2 min. To measure the activity of dehydroascorbate reductase (DHAR; EC 1.8.5.1), the method of Nakano and Asada (1981) was followed and the absorbance taken at 265 nm for 2 min. The method described by Hossain et al. (1984) was used to assay the activity of monodehydroascorbate reductase (MDHAR; EC 1.6.5.4) and the absorbance taken for 2 min at 340 nm. On the other hand, the method of Mukherjee and Choudhuri (1983) was used for the estimation of the ascorbate (AsA) content, while Ellman (1959) method was followed for the estimation of the content of reduced glutathione (GSH). Standard curves of AsA and GSH were used for their calculation.

### Estimation of ions

The concentrations of Mg, K, and Ni were determined using an atomic absorption spectrophotometer (AA-6300; Shimadzu, Kyoto, Japan) (Sagner et al., 1998). The nitrogen content was estimated in dry plant tissue according to the micro-Kjeldahl method (Jackson, 1973), while phosphorus was estimated using the spectrophotometric method (Olsen et al., 1954).

### Statistical analysis

The values presented are the mean ± SE of four replicates. Duncan’s multiple range test was performed using one-way ANOVA to determine the least significant difference (LSD) among the mean values at *p* < 0.05.

## Results

### Exogenous NO and/or Spd improves growth

The impacts of Ni stress on plant height and plant dry weight with and without the application of NO and Spd are shown in **Table 1**. Relative to the control, Ni treatment reduced the plant height (30.39%) and plant dry weight (41.50%) significantly. However, the application of NO and Spd significantly mitigated the decline in plant height and plant dry weight, with maximal ameliorations of 42.44% and 60.58% in Ni+NO+Spd-treated plants compared to their Ni-stressed counterparts. Under normal conditions, the application of NO and Spd considerably enhanced the plant height and dry weight, attaining maximal increases of 46.30% and 25.89%, respectively, due to the combined of NO and Spd treatment (**Table 1**).

**Table 1:** Effect of nitric oxide (NO; 50 µM) and spermidine (Spd; 100 µM) supplementation on nitrogen (N), phosphorous (P), potassium (K), magnesium (Mg), nickel (Ni), proline, glycine betaine, plant height, and plant dry weight in *Solanum lycopersicum* L. treated with nickel (Ni).

	Control	Spd	NO	Spd+NO	Ni	Ni+Spd	Ni+NO	Ni+Spd+NO
N	17.02 ± 1.31d	21.67 ± 1.61c	24.03 ± 1.82b	26.44 ± 2.3a	7.15 ± 0.37g	9.23 ± 0.52f	12.97 ± 0.62e	15.98 ± 1.01d
P	14.03 ± 0.99d	17.06 ± 1.17c	19.11 ± 1.32b	21.86 ± 2.1a	6.16 ± 0.31g	8.71 ± 0.49f	10.67 ± 0.81e	13.23 ± 0.79d
K	16.09 ± 0.83d	18.36 ± 1.36c	22.12 ± 1.62b	24.50 ± 1.82a	8.13 ± 0.62g	10.03 ± 0.88f	12.44 ± 0.99e	15.42 ± 1.17d
Mg	8.12 ± 0.45d	9.55 ± 0.63c	11.02 ± 0.77b	12.98 ± 0.83a	4.66 ± 0.23g	5.44 ± 0.25f	6.13 ± 0.28e	7.82 ± 0.33d
Ni	0.0024 ± 0.00019g	0.0017 ± 0.00016f	0.0013 ± 0.00011e	0.0009 ± 0.0001e	3.11 ± 0.21a	2.39 ± 0.18b	2.01 ± 0.13c	1.55 ± 0.011d
Proline	42.1 ± 3.4f	63.8 ± 4.5d	72.7 ± 5.4c	85.1 ± 5.70b	52.2 ± 3.8e	73.1 ± 5.6c	86.5 ± 6.1b	101.4 ± 6.4a
Glycine betaine	2.08 ± 0.11f	2.93 ± 0.16d	3.29 ± 0.19c	3.58 ± 0.19b	2.61 ± 0.21e	3.34 ± 0.23c	3.63 ± 0.25b	4.32 ± 0.28a
Plant height	35.2 ± 2.8d	39.5 ± 3.2c	44.3 ± 3.6b	51.5 ± 4.1a	24.5 ± 2.1g	27.7 ± 2.4f	30.3 ± 2.5e	34.9 ± 2.5d
Plant dry weight	4.12 ± 0.24d	4.73 ± 0.27c	5.17 ± 0.31b	5.56 ± 0.34a	2.41 ± 0.15g	2.98 ± 0.17f	3.36 ± 0.19e	3.87 ± 0.21d

Data are the mean ± SE of four replicates. Different letters designate significant difference at p < 0.05.

### NO and/or Spd alleviates the decline in nutrient uptake and reduced the Ni content

Nickel toxicity resulted in a significant decline in the uptake of essential elements, including N, P, K, and Mg; however, the application of NO and Spd, individually and in combination, increased the uptake of these elements. Relative to the control, Ni toxicity reduced N by 57.99%, P by 56.09%, K by 49.47%, and Mg by 42.61%, but significantly increased Ni (**Table 1**). Treatment with NO+Spd in Ni-stressed plants maximally alleviated the decreases in N (123.49%), P (114.77%), K (89.66%), and Mg (67.81%) compared to Ni-treated plants. Under normal conditions, the uptake of N, P, K, and Mg increased maximally by 55.34%, 55.80%, 52.26%, and 59.85% due to NO+Spd treatment. The application of Spd, NO, and Spd+NO to Ni-treated plants reduced the Ni accumulation by 23.15%, 35.36%, and 50.16%, respectively, compared to the Ni-stressed plants (**Table 1**).

### Proline and glycine betaine accumulation increased due to NO and Spd treatments

Plants treated with Ni exhibited increased accumulation of proline (23.99%) and GB (25.48%) compared to the control. The contents of proline and GB further increased due to the application of Spd and NO, attaining maximal increases of 140.85% and 107.69%, respectively, in Ni+Spd+NO-treated plants compared to the control. Under normal conditions, increases of 51.54% and 29.01% due to Spd, 72.68% and 58.17% due to NO, and 102.13% and 72.11% due to Spd+NO supplementation were observed compared to the control (**Table 1**).

### Supplementation of NO and/or Spd improves pigment synthesis

**Figure 1:**
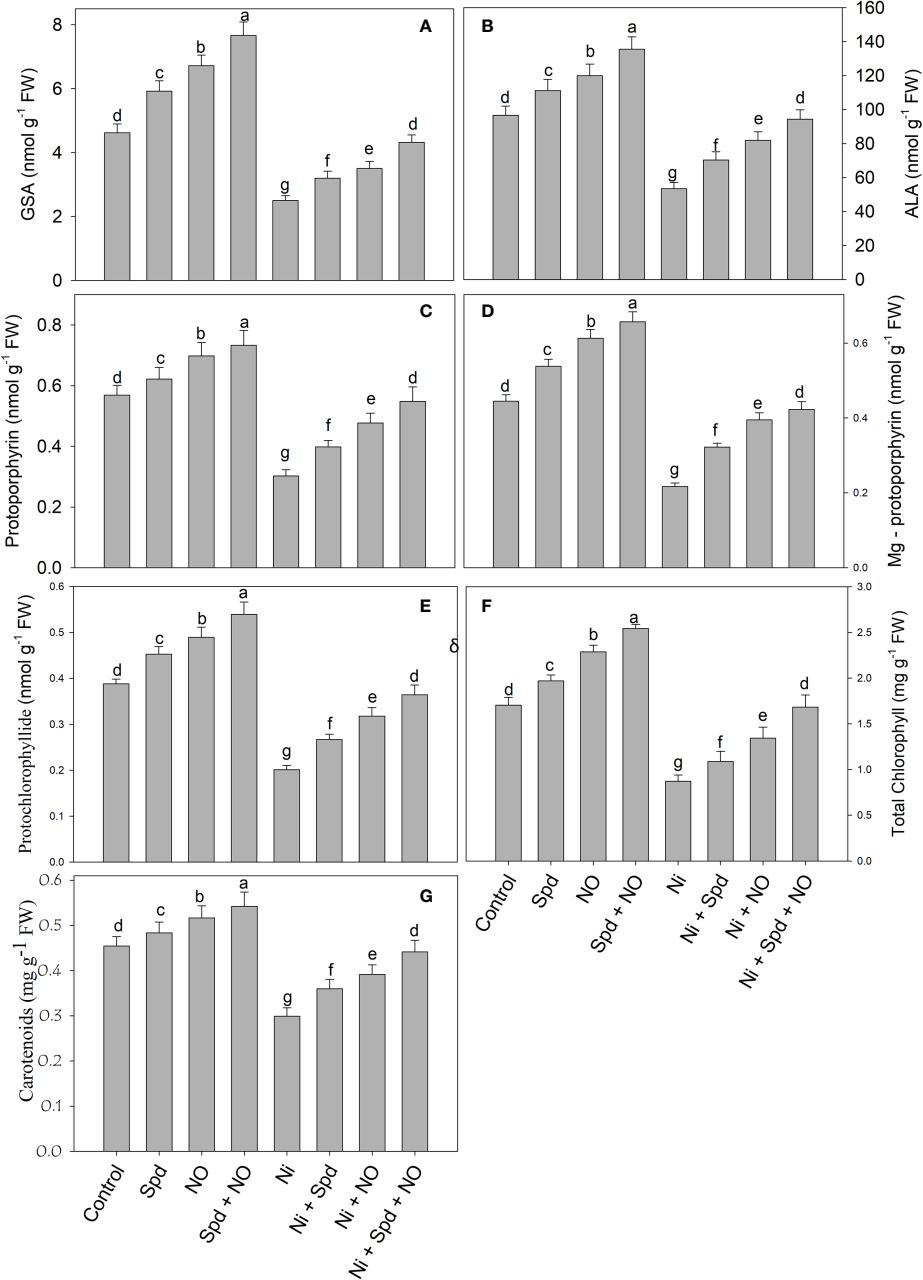
Effect of nitric oxide (50 µM) and spermidine (100 µM) supplementation on **(A)** glutamate 1-semialdehyde, **(B)** δ-amino levulinic acid, **(C)** prototoporphyrin IX, **(D)** Mg-prototoporphyrin IX, **(E)** protochlorophyllide (Pchlide), **(F)** total chlorophyll and **(G)** carotenoids content in *Solanum lycopersicum* L. subjected to nickel stress. Data is mean (±SE) of four replicates and different letters on bars denote significant difference at P < 0.05.

Treatment of tomato with Ni resulted in a significant decline in the synthesis of GSA (45.02%), δ-ALA (44.72%), Proto IX (46.92%), Mg-Proto IX (51.23%), Pchlide (48.20%), total chlorophyll (48.73%), and carotenoids (34.17%). Relative to the control, treatment with Spd+NO increased GSA by 65.80%, δ-ALA by 40.26%, Proto IX by 28.82%, Mg-Proto IX by 47.64%, Pchlide by 39.00%, total chlorophyll by 49.26%, and carotenoids by 19.30%. Alleviation of the negative effects of Ni toxicity due to individual and combined Spd and NO treatments was observed. Maximal mitigation rates of 70.07% for GSA, 76.59% for δ-ALA, 81.45% for Proto IX, 94.93% for Mg-Proto IX, 81.38% for Pchlide, 92.66% for total chlorophyll, and 47.54% for carotenoids in Ni+Spd+NO-treated compared to Ni-treated plants were observed (**Figures 1A** **–G**) .

### NO and/or Spd alleviates the decline in gas exchange and fluorescence parameters

**Figure 2:**
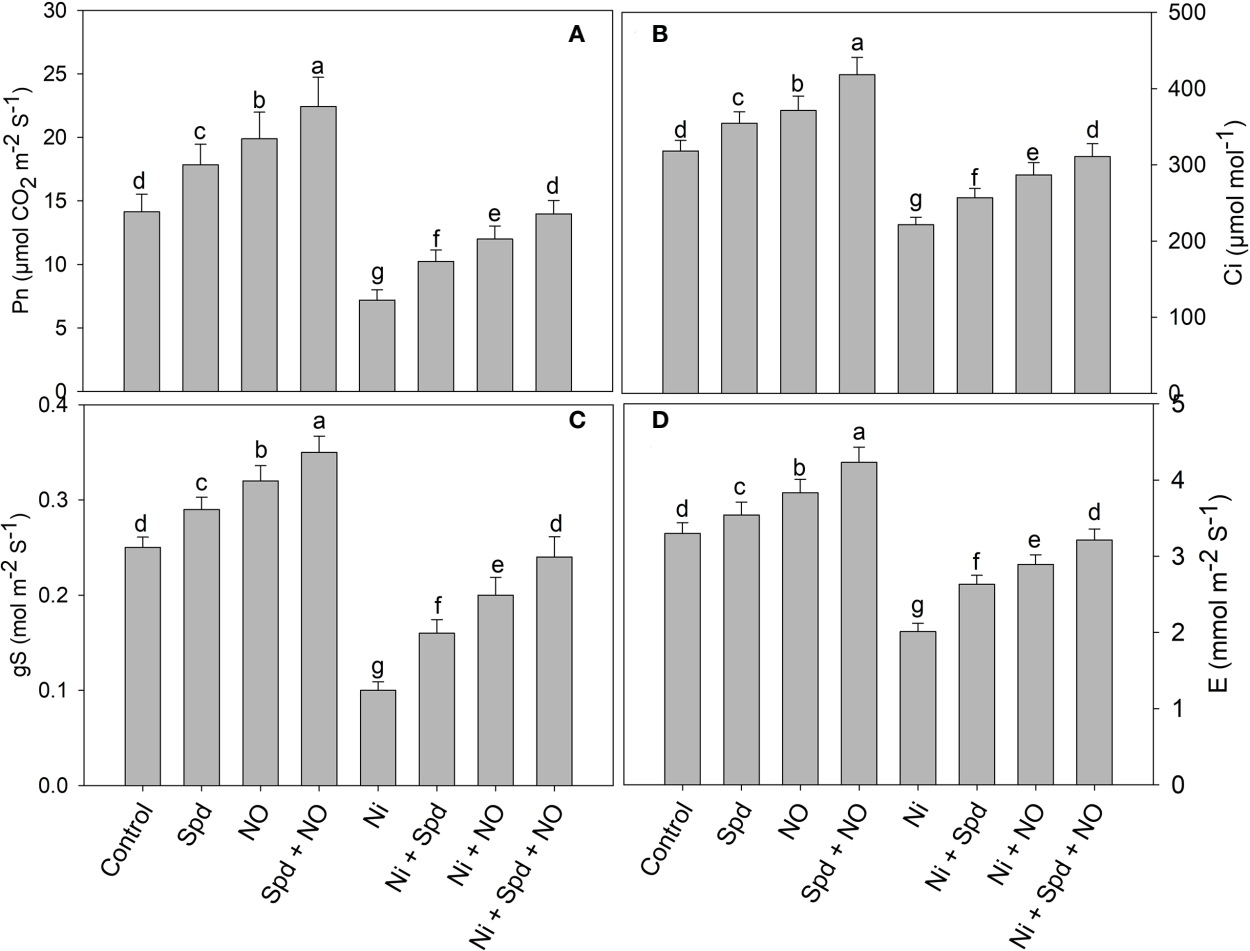
Effect of nitric oxide (50 µM) and spermidine (100 µM) supplementation on **(A)** photosynthesis, **(B)** intercellular CO2 concentration, **(C)** stomatal conductance and **(D)** transpiration rate in *Solanum lycopersicum* L. subjected to nickel stress. Data is mean (±SE) of four replicates and different letters on bars denote significant difference at P < 0.05.

**Figure 3:**
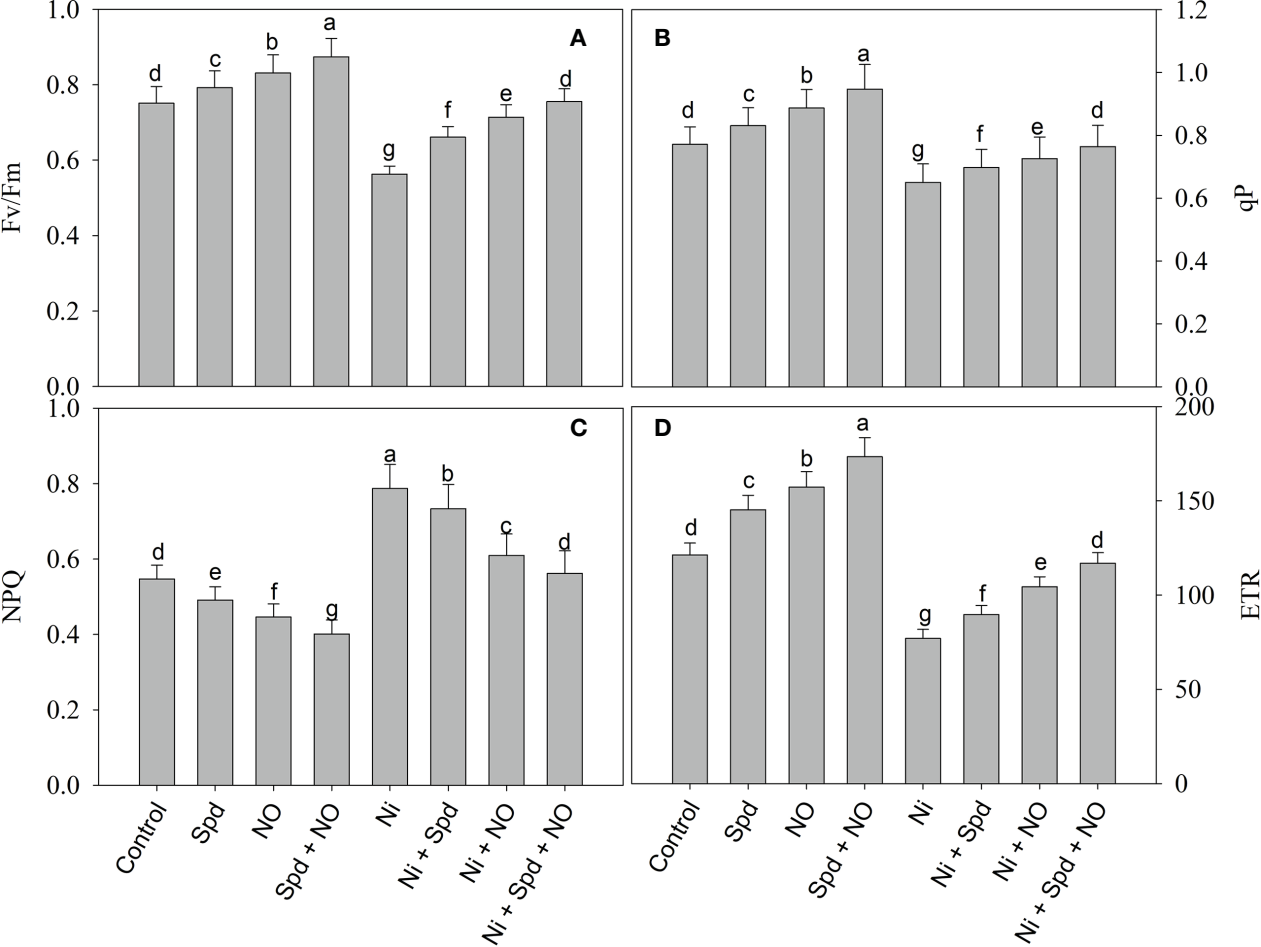
Effect of nitric oxide (50 µM) and spermidine (100 µM) supplementation on **(A)** PSII activity (Fv/Fm), **(B)** photochemical quenching (qP), **(C)** non photochemical quenching (NPQ) and **(D)** electron transport rate (ETR) in *Solanum lycopersicum* L. subjected to nickel stress. Data is mean (±SE) of four replicates and different letters on bars denote significant difference at P < 0.05.

The effects of Ni toxicity on the photosynthetic parameters *P*_n_, *E*, *C*_i_, *g*_s_, *F*_v_/*F*_m_, qP, NPQ, and ETR are shown in **Figures 2** and **3**. Relative to the control, Ni-treated plants exhibited decline rates of 49.18% in *P*_n_, 30.46% in *C*_i_, 60.00% in *g*_s_, 39.09% in *E*, 25.03% in *F*_v_/*F*_m_, 18.69% in qP, and 36.51% in ETR; however, NPQ increased by 43.87%. However, the application of NO and Spd individually and in conjunction alleviated the Ni-induced decline to a considerable extent. Under normal conditions, the combined application of Spd and NO maximally increased the *P*_n_, *C*_i_, *g*_s_, E, *F*_v_/*F*_m_, qP, and ETR by 58.74%, 31.44%, 40.00%, 28.18%, 16.37%, 22.82%, and 42.95%, respectively, compared to the control, but decreased NPQ by 47.98% (**Figures 2** and **3**).

### NO and/or Spd alleviates the oxidative damage induced by Ni

**Figure 4:**
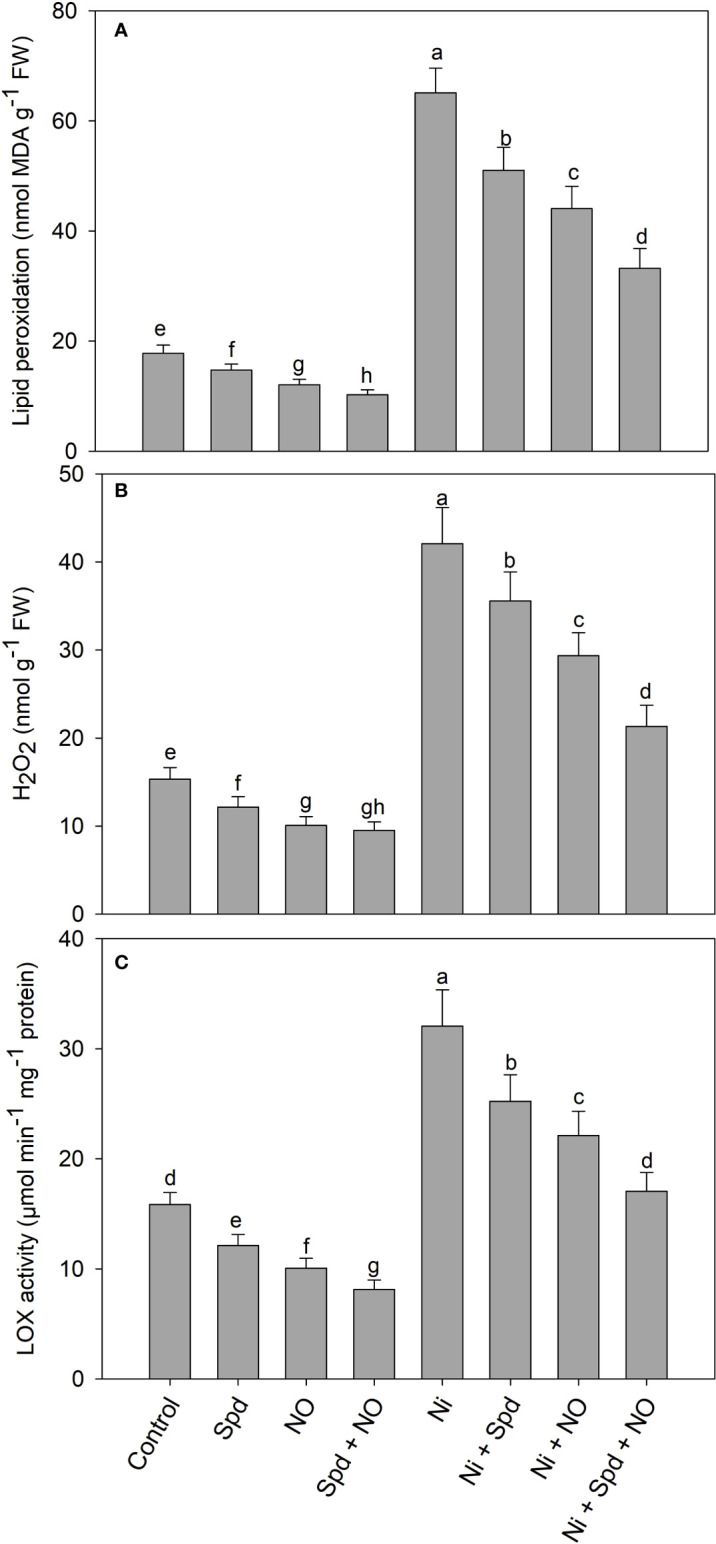
Effect of nitric oxide (50 µM) and spermidine (100 µM) supplementation on **(A)** lipid peroxidation, **(B)** hydrogen peroxide and **(C)** activity of lipoxygenase in *Solanum lycopersicum* L. subjected to nickel stress. Data is mean (±SE) of four replicates and different letters on bars denote significant difference at P < 0.05.

Treatment with Ni resulted in the increased generation of H_2_O_2_ (174.25%), the activity of lipoxygenase (102.39%), and lipid peroxidation (266.55%) compared to the control. Relative to the control, the application of Spd and NO significantly reduced H_2_O_2_, the activity of lipoxygenase, and lipid peroxidation, attaining maximal reductions of 38.13%, 48.67%, and 42.45%, respectively, in seedlings treated with Spd+NO compared to the control (**Figure 4**). Treatment with Spd and NO individually or in combination in Ni-treated plants decreased the generation of H_2_O_2_, the activity of lipoxygenase, and lipid peroxidation, with maximal reductions of 49.34%, 46.78%, and 48.98%, respectively, observed in Ni+Spd+NO-treated compared to Ni-treated plants (**Figures 4A** **–C**).

### Activity of glyoxalase system enzymes is upregulated due to NO and/or Spd

**Figure 5:**
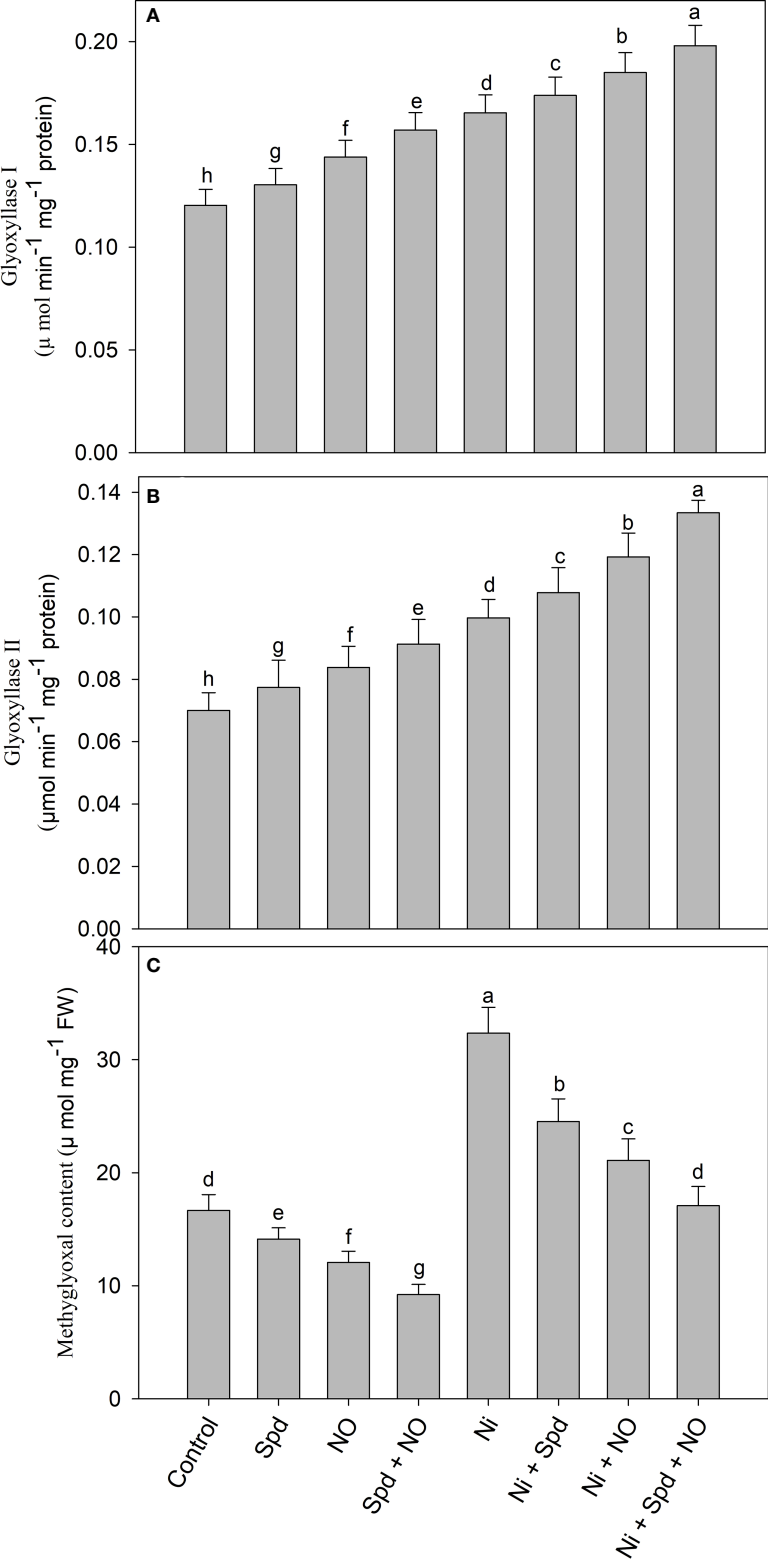
Effect of nitric oxide (50 µM) and spermidine (100 µM) supplementation on **(A)** activity of **(A)** glyoxylase I, **(B)** glyoxylase II and **(C)** methylglyoxal content in *Solanum lycopersicum* L. subjected to nickel stress. Data is mean (±SE) of four replicates and different letters on bars denote significant difference at P < 0.05.

Plants treated with Ni exhibited increased accumulation of MG and activities of Gly I and Gly II compared to the control (**Figures 5A** **–C**). Increases of 94.11% in the MG content, 37.40% in the activity of Gly I, and 42.42% in Gly II activity due to Ni toxicity were observed compared to the control. The application of Spd and NO to Ni-treated plants further increased the activities of Gly I and Gly II, attaining maximal increases of 65.00% and 90.57%, respectively, in Ni+Spd+NO-treated plants compared to the control. Under normal conditions, the application of Spd and NO increased the activities of Gly I and Gly II compared to the control, with increases of 30.42% and 31.41%, respectively, in plants treated with Spd+NO. The content of MG exhibited decline rates of 15.24%, 27.61%, and 44.65% due to Spd, NO, and NO+Spd treatments, respectively, compared to the control. The application of Spd and NO individually or in combination to Ni-treated plants reduced the MG content significantly compared to Ni-stressed plants, attaining a maximal decline rate of 47.18% in Ni+Spd+NO-treated plants compared to Ni-treated plants (**Figure 5**).

### Influence of NO and/or Spd on the antioxidant system

The activities of SOD, APX, MDHAR, DHAR, and GR increased by 92.58%, 37.91%, 84.39%, 65.74%, and 49.59%, respectively, due to Ni toxicity compared to the control. The application of Spd and NO to Ni-stressed plants caused a further increase in their activities, which showed maximal enhancements of 196.00%, 140.82%, 146.47%, 132.50%, and 160.48% for SOD, APX, MDHAR, DHAR, and GR, respectively in Ni+Spd+NO-treated plants compared to the control (**Table 2**). Under normal growth conditions, Spd and NO treatments enhanced the activities of the antioxidant enzymes assayed; however, maximal increases of 66.86% for SOD, 29.28% for APX, 61.48% for MDHAR, 49.70% for DHAR, and 50.80% for GR were exhibited by plants that received treatment with Spd+NO compared to the control. The contents of AsA and GSH respectively increased by 11.28% and 12.69% due to Spd, by 18.61% and 20.36% due to NO, and by 30.17% and 23.75 due to Spd+NO treatments compared to the control. Relative to the control, Ni treatment reduced AsA by 27.61%, but increased GSH by 22.81%. The application of Spd and NO alleviated the decline in AsA, with a maximum alleviation of 35.42% in Ni+Spd+NO-treated plants compared to their Ni-stressed counterparts. The content of GSH further increased due to the application of Spd and NO, gaining a maximum increase of 49.42% in Ni+Spd+NO-treated plants compared to the control (**Table 2**).

**Table 2:** Effect of nitric oxide (NO; 50 µM) and spermidine (Spd; 100 µM) supplementation on the activity of superoxide dismutase (SOD), ascorbate peroxidase (APX), monodehydroascorbate reductase (MDHAR), dehydroascorbate reductase (DHAR), and glutathione reductase (GR) and the contents of ascorbate (AsA) and reduced glutathione (GSH) in *Solanum lycopersicum* L. treated with nickel (Ni).

	Control	Spd	NO	Spd+NO	Ni	Ni+Spd	Ni+NO	Ni+Spd+NO
SOD	12.01 ± 0.917h	15.12 ± 1.27g	17.53 ± 1.58f	20.04 ± 1.91e	23.13 ± 2.32d	28.87 ± 2.52c	31.21 ± 2.88b	35.55 ± 3.24a
APX	0.823 ± 0.063h	0.907 ± 0.068g	0.961 ± 0.073f	1.064 ± 0.087e	1.135 ± 0.091d	1.491 ± 0.098c	1.709 ± 0.097b	1.982 ± 0.10a
MDHAR	32.04 ± 2.7h	37.21 ± 3.2g	43.06 ± 3.7f	51.74 ± 4.2e	59.08 ± 4.6d	65.15 ± 4.8c	70.83 ± 5.2b	78.97 ± 5.5a
DHAR	51.44 ± 3.7h	60.28 ± 4.5g	68.06 ± 4.7f	77.01 ± 4.8e	85.26 ± 5.3d	94.8 ± 5.8c	107.51 ± 6.1b	119.6 ± 6.4a
GR	1.24 ± 0.061h	1.34 ± .065g	1.51 ± 0.069f	1.87 ± 0.077e	2.46 ± 0.82d	2.73 ± 0.087c	2.96 ± 0.091b	3.23 ± 0.11a
AsA	333.1 ± 13.2d	370.7 ± 16.6c	395.1 ± 18.6b	433.6 ± 20.1a	241.1 ± 9.2g	278.7 ± 10.1f	298.8 ± 11.6e	326.5 ± 13.0d
GSH	319.1 ± 11.3h	359.6 ± 13.5g	384.1 ± 15.7f	418.5 ± 17.8e	391.9 ± 16.6d	436.3 ± 20.3c	456.8 ± 20.8b	476.8 ± 21.2a

Data are the mean ± SE of three replicates. Different letters designate significant difference at p < 0.05.

## Discussion

Metal pollution is one of the primary problems impeding sustainable crop production. Metals and metalloids hinder growth by causing alterations in root development, enzyme activity, photosynthesis, and floral development (Hameed et al., 2016; Alyemeni et al., 2018). Management techniques and biotechnological approaches have been adopted to strengthen the tolerance of plants to the toxic effects of metals so that the damage to productivity can be lessened. In the present study, an effort was made to investigate the beneficial role of the application of NO and Spd for the protection of tomato from the damaging effects of excess Ni. The application of NO and Spd was proven beneficial in improving growth in terms of plant height and dry biomass accumulation under normal conditions, which also alleviated the decline induced by Ni; however, the effect was more obvious in plants given both NO and Spd treatments. Ni-induced growth reduction has been previously reported by others (Soliman et al., 2019; Amjad et al., 2020). Treatment with Ni restricts growth by interfering with the cell division of the root and metaxylem cells, thereby restricting the cellular proliferation and tissue elongation (Demchenko et al., 2005). Treatment with NO and Spd may have regulated the cellular division and tissue proliferation (Shen et al., 2013); however, the mechanisms underlying the mitigation of Ni-induced growth retardation by NO and Spd are largely unknown. Improved growth and alleviation of Ni-induced toxic effects due to NO and Spd supplementation can be ascribed to the significant improvement in the uptake of key nutrients such as N, P, K, and Mg. Maintaining sufficient concentrations of the essential mineral nutrients triggers growth stimulation and other regulatory mechanisms through their active involvement in key functions including enzyme activity, cell division, photosynthesis, and activation of the tolerance mechanisms (Sarwar et al., 2019). For example, N (Iqbal et al., 2015), P (Begum et al., 2020), and K (Ahanger and Agarwal, 2017) have been reported to regulate growth under different stresses by upregulating the tolerance mechanisms, thereby neutralizing the damaging effects of ROS on delicate molecules and pathways. A reduction in the mineral elements caused by Ni stress has been reported by Soliman et al. (2019) and Amjad et al. (2020). Ni not only interferes with the uptake of mineral ions through the roots but also disrupts their translocation to the shoot and fruit (Pandey and Sharma, 2002). Treatment with Ni reduced the influx and translocation of essential elements, such as S, P, Mg, Ca, Zn, Fe, and Mn, in different crops (Yang et al., 2008). The exogenous application of NO and Spd resulted in a significant increase in the uptake of N, P, K, and Mg, but reduced the uptake of Ni. The application of NO to tomato plants has been reported to alleviate the decrease in the uptake of essential elements under Cd stress (Ahmad et al., 2018). In rice, supplementation of Spd alleviated the decrease in Mg, K, Ca, Fe, Mo, and Mn under Al stress, thereby preventing the growth retardation and photosynthetic decline (Jiang et al., 2021). In corroboration with our findings, Kotapati et al. (2017) have also demonstrated an alleviation of the decrease in the growth and mineral uptake of *Eleusine coracana* subjected to Ni stress with exogenous application of NO. However, there are no reports on the combined effect of NO and Spd on the alleviation of the Ni-induced decline in growth and mineral uptake.

Furthermore, the increased growth due to NO and Spd treatments under normal and Ni stress conditions can be attributed to the significant enhancement in the chlorophyll synthesis and photosynthesis. The contents of intermediates of the chlorophyll synthesis pathway, i.e., GSA, δ-ALA, Proto IX, Mg-Proto IX, and Pchlide, were significantly enhanced by the application of NO and Spd, thereby causing an evident increase in chlorophyll synthesis. Environmental stresses impart deleterious effects on the synthesis of chlorophyll by hindering the synthesis of intermediate compounds (Dalal and Tripathy, 2012). Qin et al. (2020) have demonstrated that salinity considerably decreases the expression of genes coding for enzymes that catalyze the key step in the chlorophyll biosynthesis pathway, thereby affecting the synthesis of chlorophyll and its intermediate compounds. Previously, a significant reduction in the chlorophyll content of *Phaseolus vulgaris* (Taibi et al., 2016) and wheat (Parlak, 2016) due to Ni toxicity has been reported; however, there are no available reports on the influence of Ni on the contents of intermediate compounds of the chlorophyll synthesis pathways. Supplementation of NO and Spd improved the uptake of Mg, a key component of the chlorophyll molecule, and, in addition, may have upregulated the activity of enzymes that mediate the synthesis of chlorophyll. Supplementation of NO (Ahmad et al., 2020) and Spd (Li et al., 2018) has been reported to alleviate the stress-induced decrease in chlorophyll synthesis, resulting in a significant enhancement in photosynthesis and gas exchange. Carotenoids act as accessory light-harvesting pigments and protect the photosynthetic apparatus from the toxic effects of radicals, act as redox intermediates in the secondary pathway of electron transfer with PSII, and bring about the stabilization of pigment–protein complexes (Frank and Brudvig, 2004; Hashimoto et al., 2016; Zulfiqar et al., 2021). Recently, Al-Mushhin (2022) has demonstrated significant alleviation in the salinity-induced decrease in δ-ALA, Proto IX, Mg-Proto IX, chlorophyll, and photosynthesis with Spd application. The combined application of NO and Spd maximally enhanced the chlorophyll and carotenoid synthesis and the stomatal and non-stomatal attributes of photosynthesis under normal conditions and Ni treatment. The enhanced chlorophyll synthesis, photosynthesis, and fluorescence parameters due to NO and Spd treatments may be due to the significant improvement in antioxidant function resulting in the quick elimination of toxic ROS, thereby protecting the major structures of the photosynthetic apparatus. Similar to our observations, Khan and Khan (2014); Soliman et al. (2019), and Khan et al. (2016) have reported significant decreases in the stomatal (*P*_n_, *C*_i_, *g*_s_, and *E*) and non-stomatal (*F*_v_/*F*_m_, qP, and ETR) attributes of photosynthesis due to Ni toxicity. Both NO and Spd have been reported to impart beneficial effects on the photosynthetic efficiency by regulating the gas exchange, water uptake, and the PSII function (Li et al., 2015; Ahmad et al., 2018; Ahmad et al., 2020). Feng et al. (2021) have demonstrated that treatment with NO induces photoprotection of PSI and PSII by initiating the D1 protein repair pathway under cold stress. Similarly, Spd-induced protection to D1 protein under salinity–alkalinity stress has been reported by Hu et al. (2016). Recently, in heat-stressed lettuce, He et al. (2022) have also observed significant alleviation in the parameters *P*_n_, *C*_i_, *g*_s_, *E*, *F*_v_/*F*_m_, qP, and ETR with Spd application, which resulted in improved growth, water use efficiency, and biomass production. Improved chlorophyll synthesis and photosynthesis in NO- and Spd-treated plants can directly influence the carbon metabolism and the carbon–nitrogen balance (Du et al., 2016; Farag et al., 2017), which will otherwise be drastically affected by Ni stress (Sheoran et al., 1990). NO and Spd might have imparted synergistic effects to protect photosynthesis. Further studies are of interest.

In addition to the growth enhancement and photoprotection, the application of NO and Spd induced a significant reduction in the oxidative damage by reducing the generation of toxic radicals such as H_2_O_2_. Excess concentrations of ROS are toxic to normal plant metabolism and cause membrane dysfunction due to the oxidation of membrane lipids and proteins, thereby affecting the functions of key organelles such as chloroplast and the mitochondria (Shahid et al., 2014; Kapoor et al., 2019). An increased ROS production and lipid peroxidation due to Ni treatment has been reported in *Brassica juncea* (Khan et al., 2016), *Zea mays* (Amjad et al., 2020), and *Vicia faba* (Helaoui et al., 2022). Increased ROS due to stress conditions triggers the peroxidation of membrane lipids and proteins, thereby reducing their structural and functional stability, causing the leakage of essential cellular constituents to occur (Ahanger et al., 2019; Qin et al., 2021). Exogenous application of NO (Fatma et al., 2016) and Spd (Li et al., 2015) significantly reduces the generation of ROS, hence protecting the growth and photosynthetic function by maintaining the structural and functional stability of the key components. However, the combined effect of NO and Spd has not been investigated. Increased lipoxygenase activity determines the enhanced hydroperoxidation of polyunsaturated fatty acids (Babenko et al., 2017), and elevated activity of lipoxygenase reflects a surge in damage to membrane fatty acids (Nahar et al., 2016). The reduced accumulation of ROS in NO- and Spd-treated plants can be due to the significant upregulation of the antioxidant system, which reduces the peroxidation of membranes, hence protecting the function of the major cellular organelles. SOD forms the key defense against superoxide radicals, while APX, MDHAR, DHAR, and GR are important enzymatic components of the ascorbate–glutathione cycle, which also involves AsA and GSH (Ahanger et al., 2017; Kapoor et al., 2019). Although Ni toxicity triggered the activity of antioxidant enzymes, the application of NO and Spd further increased their activity, thereby strengthening ROS scavenging. Spd (Jiang et al., 2021) and NO (Fatma et al., 2016) treatment has previously been reported to upregulate the antioxidant function under aluminium and salt stress, thereby reducing the ROS accumulation and lipid peroxidation. The upregulation of the antioxidant system leads to the regulation of growth, membrane function, redox homeostasis, photosynthesis, and mitochondrial electron transport (Ahmad et al., 2018). The ascorbate–glutathione cycle eliminates excess H_2_O_2_ from the chloroplast and the mitochondria and maintains the optimal concentrations of the redox components, including AsA and GSH (Hasanuzzaman et al., 2019b). GSH has an important role in glyoxalase cycle as well. In addition, the upregulation of the ascorbate–glutathione cycle leads to the maintenance of optimal NADPH/NADP so that the electron transport is least affected and radical generation is reduced (Ahmad et al., 2020). In the present study, NO- and Spd-treated plants maintained an increased ETR and concentration of the redox components, which can be ascribed to the upregulated function of the ascorbate–glutathione cycle. Both ascorbate and glutathione are involved in the elimination of key components of the redox system and the ascorbate–glutathione cycle; therefore, their increased accumulation due to NO and Spd can eliminate the stress-induced damaging effects on the key cellular organelles, macromolecules, and pathways. By maintaining the redox state of α-tocopherol and zeaxanthin, glutathione protects the biological membranes and prevents the oxidative denaturation of proteins under stress conditions by protecting thiol groups. In addition, it acts as a substrate for the key antioxidant enzymes glutathione peroxidase and glutathione *S*-transferase (Hasanuzzaman et al., 2017). Ascorbate effectively scavenges ROS directly or indirectly, thereby playing a critical role in the tolerance to oxidative damage, besides acting as a cofactor for several enzymes (Xiao et al., 2021).

The accumulation of compatible osmolytes, including proline and GB, was enhanced with Ni treatment, and supplementation of NO and Spd further enhanced their accumulation, attaining maximal accumulation in plants treated with both NO and Spd. Both proline and GB accumulate in significant concentrations to alleviate the deleterious effects of stress on plant function (Hasanuzzaman et al., 2019a; Ali et al., 2020). GB treatment significantly reduces the oxidative effects of Ni by reducing ROS and the lipoxygenase activity and increasing the proline content in *Pennisetum typhoideum* (Xalxo et al., 2017). The accumulation of proline (Singh et al., 2012; Parlak, 2016) and GB (Soliman et al., 2019) due to Ni toxicity has been reported earlier. Accumulation of compatible osmolytes protects metabolism, protein structure, and function; maintains the cellular water content; and scavenges ROS (Ali et al., 2020; Ghosh et al., 2021). The increased accumulation of osmolytes such as proline and GB due to NO and Spd application has been reported by others, similarly resulting in the alleviation of the damaging effects of different stressors on plant growth (Fatma et al., 2016; Pal et al., 2018; Ahanger et al., 2019; Wang et al., 2020). The influence of NO and Spd individually or in combination on the alleviation of Ni toxicity has been rarely reported. The accumulation of compatible osmolytes significantly affects the antioxidant potential of plants, hence the stress tolerance potential (Jday et al., 2016). Increased accumulation of osmolytes is regulated at the gene expression level to directly affect the function of the enzymes controlling their metabolism (Meena et al., 2019). Osmolytes such as proline, amino acids, sugars, and GB have been suggested to maintain redox homeostasis and mediate stress signaling to protect key plant functions including photosynthesis (Rosa et al., 2009; Kishor et al., 2015; Trovato et al., 2021; Kishor et al., 2022). Therefore, the increased accumulation of osmolytes due to NO and Spd treatment under Ni stress determines their beneficial role in preventing the damaging effects on plant growth.

The glyoxalase system is another interesting mechanism to protect plants from toxic MG, and manipulating the activity of glyoxalase enzymes helps plants counter the damaging effects efficiently (Gupta et al., 2018). The activities of both Gly I and Gly II were significantly enhanced with the supplementation of NO and Spd, and the effect was more obvious due to their combined treatment. The application of NO (Ahmad et al., 2020) and Spd (Nahar et al., 2017) has been demonstrated to improve the activities of Gly I and Gly II, which was reflected in the reduced accumulation of MG, hence preventing growth retardation under arsenic and aluminium stress. The effect of NO or Spd on the function of the glyoxalase system has not been reported under Ni stress. In cadmium-stressed mung bean, Nahar et al. (2016) reported that the combined application of NO and Spd enhanced the glyoxalase function more efficiently than did individual treatments, which was reflected in the reduced MG accumulation. MG is cytotoxic at higher concentrations (Kaur et al., 2014) and can be an important signaling molecule if maintained at low concentrations (Hoque et al., 2016). Plants able to maintain the significantly increased activity of the glyoxalase system combat the stress-induced damaging effects more efficiently (Maier et al., 2012). Crop cultivars exhibiting increased activities of the antioxidant and glyoxalase system enzymes display better stress adaptation potential and strengthening of the tolerance mechanism due to exogenous protectants, proving their beneficial role in stress mitigation and their contribution toward sustainable food production (Ahmad et al., 2021).

## Conclusion

Conclusively, it can be said that exogenously supplied NO and Spd have been proven beneficial in assuaging the damaging effects of Ni on the growth, chlorophyll synthesis, and photosynthesis of tomato. The alleviatory effects of NO and Spd were evident as significant reductions in ROS and MG accumulation, lipid peroxidation, and lipoxygenase activity. Furthermore, increased osmolyte accumulation and enhanced function of the antioxidant and glyoxalase systems justify the beneficial influence of NO and Spd on Ni tolerance. The results suggest a crosstalk mechanism between NO and Spd for efficient Ni toxicity adaptation at the physiological and biochemical levels.

## Data availability statement

The original contributions presented in the study are included in the article/supplementary material. Further inquiries can be directed to the corresponding author.

## Author contributions

CQ and MA conceived and designed the study. CQ and JS carried out the experimentation. CQ, JS, and MA compiled the literature. CQ and JS wrote the initial draft. MA crosschecked the results and revised the manuscript. All authors contributed to the article and approved the submitted version.

## Funding

This work was supported by the Natural Science Foundation for Young Scientists of Shanxi Province (nos. 20210302124362 and 20210302124506) and the Shanxi Province Higher Education Science and Technology Innovation Program Project (no. 2021L510).

## Conflict of interest

The authors declare that the research was conducted in the absence of any commercial or financial relationships that could be construed as a potential conflict of interest.

## Publisher’s note

All claims expressed in this article are solely those of the authors and do not necessarily represent those of their affiliated organizations, or those of the publisher, the editors and the reviewers. Any product that may be evaluated in this article, or claim that may be made by its manufacturer, is not guaranteed or endorsed by the publisher.
