# Corrigendum: Supplementation of nitric oxide and spermidine alleviates the nickel stress induced damage to growth, chlorophyll metabolism and photosynthesis by up-regulating ascorbate-glutathione and glyoxalase cycle functioning in tomato

**DOI:** 10.3389/fpls.2023.1239602

**Published:** 2023-07-25

**Authors:** Cheng Qin, Jie Shen, Mohammad Abass Ahanger

**Affiliations:** ^1^Department of Life Sciences, University of Changzhi, Changzhi, China; ^2^College of Life Science, Northwest A&F University, Xianyang, Shaanxi, China

**Keywords:** antioxidants, glyoxalase, oxidative stress, nickel, nitric oxide, spermidine

In the published article, there was an error in the **Funding** statement. The grant number for funding from Shanxi Province Higher Education Science and Technology Innovation Program Project was incorrectly reported as “no. 2021L511”.

The correct **Funding** statement appears below.

“This work was supported by the Natural Science Foundation for Young Scientists of Shanxi Province (nos. 20210302124362 and 20210302124506) and the Shanxi Province Higher Education Science and Technology Innovation Program Project (no. 2021L510).”

The authors apologize for this error and state that this does not change the scientific conclusions of the article in any way. The original article has been updated.

